# Neuropharmacological Interventions of Plant Origin for Parkinson's Disease: A Comprehensive Appraisal

**DOI:** 10.2174/1570159X23666250523112027

**Published:** 2025-05-26

**Authors:** Umar Muzaffer, Bisma Gull, Zabeer Ahmed, Muzamil Ahmad

**Affiliations:** 1 Department of Medicine, Govt, Medical College and Associated SMHS Hospital Srinagar, J&K-190001, India;; 2 Pharmacology Division, CSIR-Indian Institute of Integrative Medicine, Sanat Nagar, Srinagar, Jammu and Kashmir, J&K-190005, India;; 3 Pharmacology Division, CSIR-Indian Institute of Integrative Medicine, Jammu Tawi, Jammu and Kashmir, J&K-180001, India

**Keywords:** Parkinson's disease, neuropharmacology, geriatric psychiatry, ethnopharmacology, natural compounds, synergistic effects

## Abstract

Parkinson's disease (PD) presents a complex challenge in neurodegenerative disorders, necessitating innovative therapeutic approaches. This review article elucidates the therapeutic potential of traditional herbal formulations alongside computational methods in PD research. Through comprehensive examination, we explore their mechanisms of action, synergistic effects, and implications for PD management. Furthermore, we discuss the significance of computational techniques such as molecular docking, molecular dynamics simulations, pharmacophore modeling, and network pharmacology. Our analysis underscores the integration of traditional wisdom with modern scientific inquiry, paving the way for nuanced interventions in PD.

## 1 INTRODUCTION

Psychiatric comorbidities are frequently observed in various neurological disorders, with Parkinson's disease (PD) being a notable example. Among these comorbidities, depression, anxiety disorders, and cognitive impairment are the most prevalent in individuals with PD [1]. The clinical presentation of psychiatric symptoms often corresponds to the underlying neuropathological mechanisms of PD [2]. However, superficial evaluations may lead to misdiagnoses or inappropriate treatment strategies [3].

Despite the availability of pharmacological and surgical interventions that effectively mitigate the motor symptoms of PD, the disease inevitably progresses, leading to increased disability in most patients [4]. In the advanced stages of PD, patients often experience motor complications, including fluctuations in motor responses to medication and dyskinesia. As the disease progresses, certain symptoms become refractory to levodopa, the gold standard treatment [5, 6]. These refractory symptoms include postural instability, speech and swallowing difficulties, and a spectrum of non-motor symptoms (NMS) [6]. The complex interplay between neurological and psychiatric dimensions in PD necessitates a comprehensive evaluation and individualized treatment strategies. Inadequate recognition and understanding of these complexities can result in diagnostic errors and suboptimal management, highlighting the need for a holistic approach that addresses both the neurological and psychiatric components of the disorder. Interdisciplinary collaboration among neurologists, psychiatrists, and other healthcare professionals is crucial to improving patient outcomes as our understanding of these relationships deepens [7, 8].

Effective neuropharmacological interventions are essential for managing both motor and non-motor symptoms in PD. These interventions target dysregulated neurotransmitter activity and dysfunctional neural pathways, alleviating motor symptoms such as tremors and rigidity [9, 10]. Additionally, they address cognitive, emotional, and autonomic symptoms, thereby enhancing the overall quality of life for patients. Addressing both motor and non-motor symptoms acknowledges the multifaceted nature of PD and facilitates comprehensive patient care [11, 12]. This review article aims to elucidate the relationship between PD and psychiatric comorbidities, with a specific focus on the interplay between neurological and psychiatric symptoms. It emphasizes the importance of effective neuropharmacological interventions for managing the broad spectrum of PD symptoms. The scope of the article encompasses diagnostic challenges and underscores the necessity of a holistic, interdisciplinary approach. Ultimately, it seeks to provide a comprehensive resource for healthcare professionals, promoting better patient outcomes through integrated care strategies.

## 2 METHODOLOGY

The literature search encompassed databases including PubMed, Scopus, and Google Scholar. Search terms comprised variations of “Parkinson's disease”, “traditional herbal formulations,” “*Mucuna pruriens*”, “*Bacopa monnieri*,” and “computational methods”. Studies were selected based on relevance to PD management, herbal therapeutics, and computational approaches. Analysis methods included qualitative synthesis and critical evaluation of findings to elucidate therapeutic mechanisms and potential synergies.

## 3 PATHOPHYSIOLOGY OF PD

PD is a complex neurodegenerative disorder marked by the progressive loss of dopamine-producing neurons in the substantia nigra region of the brain. The development of PD is a complex interplay of genetic susceptibility, environmental factors, protein aggregation, mitochondrial dysfunction, and neuroinflammatory processes [13]. The progressive loss of dopamine-producing neurons and the disruption of brain circuits underlie PD's motor and non-motor symptoms. Ongoing research aims to unravel these intricate mechanisms to develop more targeted therapies for this debilitating condition [14].

PD is characterized by a significant reduction in striatal dopamine levels, a neurotransmitter critical for motor control. Dopamine, an imperative neurotransmitter, exercises profound regulatory control over motor coordination, emotional states, reward systems, and assorted cognitive processes [15]. This degeneration is chiefly observed in neurons that synthesize dopamine within the substantia nigra, ultimately culminating in an insufficiency of dopamine within the intricate neural circuits governing motor functions [16, 17]. This deficiency yields the distinctive motor symptoms characteristic of PD, encompassing tremors, muscular rigidity, bradykinesia (sluggishness in movement), and compromised postural stability. A pivotal pathological hallmark underscoring PD is the accumulation of anomalous protein aggregates, predominantly of α-synuclein, transpiring within neuronal confines. These aggregations, colloquially referred to as Lewy bodies, perturb fundamental cellular operations and intercede in the process of neuronal deterioration [18-20]. The precise mechanisms underpinning α-synuclein's aggregation and ensuing toxicity remain partially elucidated.

Nevertheless, prevailing hypotheses posit that aberrations in protein folding, clearance mechanisms, and intracellular trafficking collectively contribute to the intricate genesis of α-synuclein aggregation and its concomitant detriment. Mitochondria, esteemed as the dynamic organelles orchestrating cellular energy production, substantiate their pivotal role in the pathogenesis of PD [21, 22]. Their compromised functionality begets a milieu of perturbed processes, including heightened oxidative stress and attenuated energy generation, both profoundly implicated in the demise of neuronal populations. Neurons within the substantia nigra are particularly vulnerable to mitochondrial dysfunction due to their disproportionately high energy demands required to maintain physiological homeostasis. This intricate interplay of mechanisms, encompassing dopamine depletion, α-synuclein aggregation, and mitochondrial dysfunction, collectively underpins the intricate trajectory of PD pathogenesis. As we delve deeper into the intricate landscape of neurobiology, untangling the enigmatic threads of these mechanisms holds the promise of unearthing novel therapeutic strategies to alleviate the burdensome impact of PD on affected individuals [17, 23, 24].

Neuroinflammatory cascades intricately contribute to the progression of PD. The pivotal participants in this process are microglia, the brain's resident immune cells. In response to pathological stimuli, these microglia become activated, initiating a series of events that entail the release of pro-inflammatory molecules [25]. These molecules, including cytokines, chemokines, and reactive oxygen species, wield a dual-edged impact. While their primary role is to combat potential threats, their immoderate release can inadvertently inflict collateral damage on neurons, thus amplifying the degenerative trajectory [26, 27]. This inflammatory milieu perpetuates neuronal dysfunction and aggravates the existing pathogenic cycle. The ramifications of neuroinflammation extend beyond the immediate vicinity of activated microglia.

Emerging evidence suggests that chronic neuroinflammation might foster the dissemination of α-synuclein pathology, a defining pathological hallmark of PD. The precise mechanisms dictating this propagation remain under scrutiny. However, it is postulated that the inflammatory microenvironment might engender conditions conducive to the aggregation and trans-synaptic transfer of α-synuclein aggregates, thereby propagating the pathological burden to distant brain regions. In the landscape of PD etiology, genetic influences loom prominently. Though most PD cases transpire sporadically, genetic factors yield substantial sway over disease susceptibility [28, 29]. Noteworthy genetic mutations, notably within genes like SNCA (coding for α-synuclein), LRRK2, PARKIN, PINK1, and DJ-1, have been linked to familial forms of PD These genetic aberrations elicit multifaceted disturbances, perturbing diverse cellular processes ranging from protein degradation and mitochondrial function to cellular stress responses [30]. The intricate interplay of these genetic anomalies engenders an unfavorable milieu, precipitating the neurodegenerative course observed in PD. Intriguingly, environmental factors also intersect with PD predisposition. Exposure to specific neurotoxic agents, exemplified by pesticides and heavy metals, escalates the risk of PD onset. These toxins perturb neuronal integrity through mechanisms involving oxidative stress, mitochondrial dysfunction, and protein mishandling [31]. The genetic susceptibilities amplify the effect of these environmental insults, thus intensifying the neurons' vulnerability to degeneration. Genetic propensities, environmental exposures, and complex cellular interactions, including neuroinflammation, lead to an intricate tapestry of PD pathogenesis [32]. As our comprehension of these intricate threads advances, we move closer to deciphering the intricate mechanisms underlying PD, potentially unveiling novel avenues for therapeutic interventions targeting the disease's multifaceted etiology.

**Fig. (1):**
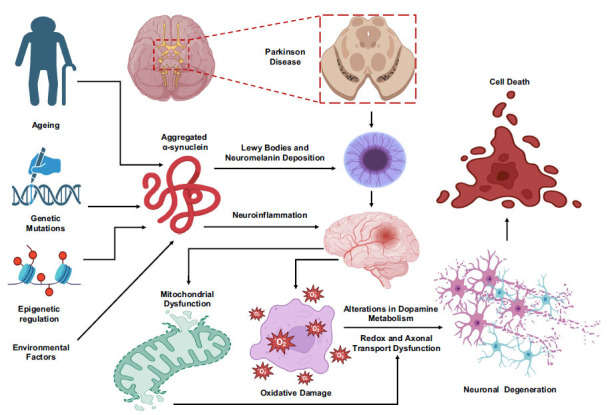
Pathophysiology of Parkinson’s disease.

Dopamine depletion stands as a hallmark event in PD progression. Dopamine, a neurotransmitter, orchestrates an array of pivotal functions, from motor regulation to mood modulation [33, 34]. In PD, the substantia nigra, a region housing dopamine-producing neurons, undergoes progressive degeneration. This loss of dopamine-producing neurons directly results in a paucity of dopamine within brain regions crucial for motor control, particularly the basal ganglia. This dearth of dopamine distorts the finely tuned balance between dopamine and other neurotransmitters, impairing the coordination of motor signals. Consequently, the characteristic motor symptoms of tremors, rigidity, bradykinesia, and postural instability emerge as a manifestation of this perturbed neural circuitry [35]. The diminution of dopamine engenders aberrant signaling patterns, precipitating the motor dysfunctions inherent to PD. Equally pivotal in the disease's trajectory is the aggregation of α-synuclein protein. α-synuclein usually plays a role in synaptic function, but in PD, it assumes an aberrant conformation that results in its accumulation within neurons, leading to the formation of Lewy bodies [36]. These protein aggregates, widely distributed in PD-affected brain regions, exert deleterious effects. The aggregated α-synuclein disrupts cellular functions, impairing synaptic transmission and protein trafficking and ultimately leading to neuronal dysfunction and degeneration [20]. The exact triggers of α-synuclein misfolding and aggregation remain an active area of investigation, with oxidative stress, impaired protein clearance mechanisms, and inflammatory responses implicated in this process. The interplay between dopamine depletion and α-synuclein aggregation is intricate. Dopamine-producing neurons are prone to α-synuclein accumulation, contributing to their vulnerability and exacerbating dopamine depletion [37, 38]. α-synuclein aggregation also sparks neuroinflammatory responses, amplifying the inflammatory environment that can further damage neurons (Fig. **1**).

Additionally, α-synuclein aggregates can disrupt mitochondrial function and increase oxidative stress, culminating in cellular damage. This interconnected web of events collectively drives disease progression. The pathogenesis of PD is not solely confined to dopamine and α-synuclein. Genetic factors, such as mutations in genes like SNCA, LRRK2, PARKIN, PINK1, and DJ-1, contribute to familial forms of PD. These genetic anomalies can potentiate α-synuclein aggregation, disrupt protein homeostasis, and impair cellular stress responses, exacerbating disease progression. Environmental toxins, including pesticides and heavy metals, also interact with these genetic susceptibilities, escalating neurodegeneration by inducing oxidative stress and mitochondrial dysfunction [36, 39].

## 4 CURRENT STANDARD OF CARE

The current standard of care for PD typically involves a combination of medications, including levodopa, dopamine agonists, and MAO-B inhibitors, tailored to manage motor symptoms while minimizing side effects. Surgical interventions like deep brain stimulation may be considered in advanced cases.

### 4.1 Levodopa

Levodopa (L-DOPA) constitutes a fundamental and extensively employed pharmacological intervention for managing PD, a neurodegenerative disorder. It serves as a precursor to dopamine, a neurotransmitter of pivotal importance in modulating movement and diverse neurophysiological functions within the brain. PD is typified by a gradual attrition of dopamine-producing neurons in the substantia nigra, a specific brain region [33]. The ensuing dopamine deficiency gives rise to a constellation of motor symptoms that characterize the ailment, including tremors, rigidity, bradykinesia (slowed movement), and disturbances in maintaining posture. Upon entering the central nervous system, Levodopa crosses the blood-brain barrier effectively and subsequently undergoes enzymatic conversion into dopamine within cerebral tissues. Notably, in contrast to dopamine, levodopa possesses the unique capability to cross the barrier mentioned above, rendering it efficacious for augmenting dopamine levels in the brain. Following its conversion into dopamine, levodopa is pivotal in compensating for the paucity of dopamine, thereby mitigating the motor symptoms accompanying PD. Its particularly pronounced efficacy lies in ameliorating the manifestations of bradykinesia and rigidity [40, 41].

The majority of individuals diagnosed with Parkinson's disease (PD) necessitate the initiation of levodopa therapy within two years from the onset of their symptoms. Levodopa, the preeminent pharmacological intervention for managing PD, is typically administered in combination with carbidopa or benserazide. These adjunct medications serve as aromatic acid decarboxylase inhibitors, thereby mitigating the peripheral metabolism of levodopa and substantially diminishing the likelihood of experiencing nausea as an adverse effect. Recent research has demonstrated that altering the carbidopa-to-levodopa ratio, moving beyond the conventional 1:4 ratio can result in an extension of the period during which the patient is free from dyskinesia (uncontrolled movements) and a reduction in off time (periods of decreased medication effectiveness).

However, the prolonged use of levodopa is associated with notable challenges. One notable hurdle is the emergence of motor fluctuations and dyskinesias over extended periods. Motor fluctuations entail alternating phases of improved motor control (“on” periods) interspersed with phases of diminished efficacy (“off” periods) as the pharmacological effects of levodopa wane between doses [42-44]. Dyskinesias, characterized by involuntary, rhythmic movements akin to dance-like motions, may manifest during these “on” periods. As PD progresses, the temporal span of levodopa's beneficial effects may contract, giving rise to the phenomenon termed “wearing-off.” In this context, Parkinsonian symptoms resurge progressively before the scheduled administration of the subsequent dose. To mitigate this effect, medical practitioners may manipulate levodopa's dosing regimen and magnitude to counteract motor fluctuations and dyskinesias.

Additionally, concurrent administration of other therapeutic agents, such as dopamine agonists, MAO-B inhibitors, and COMT inhibitors, is contemplated to enhance levodopa's efficacy while concurrently attenuating fluctuations synergistically. Incorporating extended-release formulations of levodopa is envisaged as a strategy to engender more consistent control of symptoms throughout the day, consequently ameliorating motor fluctuations [45]. In severe cases marked by symptoms refractory to treatment, administering levodopa *via* continuous infusion utilizing a specialized pump system is considered. This approach ensures a steady and sustained medication delivery, thereby minimizing fluctuations and optimizing the therapeutic benefit of levodopa [46, 47].

### 4.2 Dopamine Agonists

Dopamine agonists constitute a pharmacotherapeutic category frequently employed in the therapeutic regimen for PD [48]. Their prominent utility lies in ameliorating the motor manifestations intricately associated with the ailment, encompassing tremors, rigidity, and bradykinesia. These pharmacological agents are predicated upon their capacity to exert direct influence upon dopamine receptors within the cerebral milieu, thereby eliciting a physiological response that emulates the action of dopamine itself. This mechanism of action is of particular significance due to the diminished presence of dopamine, stemming from the progressive attrition of dopamine-producing neurons characteristic of PD [4]. Notably, dopamine agonists traverse the biological milieu to exert their modulatory effects upon receptors in the cerebral landscape. This intricate process effectuates a semblance to the action of dopamine, manifesting as the alleviation of motor symptoms plaguing individuals with PD [49, 50]. The underlying rationale for this therapeutic intervention resides in the fundamental role of dopamine as a neurotransmitter governing diverse facets of motor function, the deficiency of which underpins the motor deficits hallmarking the disease. Within the domain of dopamine agonists, two principal subdivisions emerge: the non-ergot and ergot derivatives. The former category is discernibly more prevalent and encompasses pharmacological agents characterized by their preferential utilization. It comprises substances such as pramipexole and ropinirole, which exert their effects predominantly by activating D2 and D3 dopamine receptors. These agents represent a mainstay in addressing the motor symptoms of PD, efficaciously mitigating the range of motor disturbances that ensue from the dopamine deficit [51-53].

**Fig. (2):**
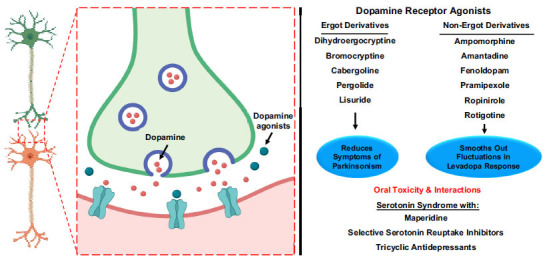
Dopamine agonists in Parkinson’s disease.

Conversely, the ergot derivatives, exemplified by bromocriptine, constitute a less commonly employed subset [54, 55]. Owing to their propensity for eliciting specific adverse effects and considerations related to their overall risk-benefit profile, ergot derivatives have become less favored in contemporary therapeutic practice. While sharing the overarching mechanism of directly stimulating dopamine receptors, these agents are distinguished by their distinct molecular characteristics and associated pharmacological outcomes [54]. In summation, dopamine agonists emerge as an integral pharmacological armamentarium in the management of PD, aptly addressing the constellation of motor deficits that are indelibly intertwined with the ailment [56]. Their therapeutic foundation rests upon their capability to engage dopamine receptors akin to endogenous dopamine, thereby rectifying the deficiency engendered by the progressive degeneration of dopamine-producing neurons. The dichotomous division into non-ergot and ergot derivatives elucidates the nuanced landscape of dopamine agonist therapy, wherein non-ergot agents, marked by their efficacy and comparatively favorable side effect profiles, assume preeminence in contemporary clinical practice [57, 58] (Fig. **2**).

#### 4.2.1 Non-ergot Dopamine Agonists

Under their propitious side effect of ergot derivatives, non-ergot dopamine agonists assume a more predominant prescription frequency. This preference stems from their efficacy in providing relief from symptoms and their extensive utilization as both primary interventions and supplementary modalities alongside other therapeutic agents in the context of PD.

Among these non-ergot agents, pramipexole is emblematic, manifesting a proclivity for stimulating D2 and D3 dopamine receptors. Pramipexole is procurable in immediate-release and extended-release formulations, conferring versatility in dosing strategies. Its deployment is often directed towards the management of incipient Parkinson's stages or as an adjunctive measure in tandem with levodopa, the objective being the amelioration of motor fluctuations encountered in the course of levodopa therapy [52, 53].

Ropinirole, another notable non-ergot dopamine agonist, evinces a predilection for activating D2 and D3 dopamine receptors. This pharmaceutical entity manifests diverse incarnations, encompassing immediate-release tablets and extended-release formulations. Ropinirole heralded for its therapeutic versatility, is employed both as monotherapy in the early stages of Parkinson's and as a complementary element within a comprehensive pharmacological regimen in the advanced disease phases [59]. In a stark departure from its precursors, Rotigotine adopts a distinctive route of administration, being available in the form of a transdermal patch that bestows a continuous supply of the medication. This unique approach ensures the maintenance of dopamine levels at equilibrium throughout the diurnal rhythm. Rotigotine influences a broad spectrum of D1, D2, D3, and D4 dopamine receptors. Its administration finds utility in diverse therapeutic scenarios, spanning the gamut from monotherapy in the nascent phases of PD to the status of an augmentative agent in the therapeutic constellation [60]. In essence, non-ergot dopamine agonists emerge as a consequential therapeutic modality in the PD landscape, lauded for their diminished proclivity for adverse effects relative to their ergot counterparts. Pramipexole, Ropinirole, and Rotigotine exemplify the trajectory of this pharmacotherapeutic domain, each embracing unique attributes and applications within the comprehensive schema of Parkinson's treatment. Distinguished by their differential receptor affinities and administration modalities, these agents collectively underscore the multifaceted approach to managing the intricate tapestry of motor symptoms that hallmark the disease.

#### 4.2.2 Ergot-Derived Dopamine Agonists

Ergot-derived dopamine agonists, having an earlier provenance, presently assume a diminished role within the therapeutic armamentarium due to their connotation with specific untoward effects, notably fibrotic complications [61]. One prototypical exemplar of this category is bromocriptine, which operates by eliciting activation of both D1 and D2 dopamine receptors. Bromocriptine, an early entrant in the armamentarium for treating PD, has witnessed receding prominence owing to the advent of contemporary non-ergot alternatives. This shift in preference stems from the concerns surrounding the propensity for adverse effects intrinsic to ergot-derived agents. Dopamine agonists serve as versatile therapeutic entities in PD management. They emerge as prime candidates for first-line therapy in specific contexts, particularly in younger cohorts with milder symptomatology. Notably, these agents confer effective symptomatic control, circumventing the immediate hazard of motor fluctuations associated with levodopa administration [62].

Furthermore, dopamine agonists play a crucial role in synergistic conjunction with other medications in the therapeutic armamentarium, notably levodopa. This therapeutic alliance, achieved through careful titration and management, elongates the duration of levodopa's efficacy while concurrently curbing instances of “off” periods and frequent dyskinesias. While efficacious, dopamine agonists are not immune to challenges. Some individuals may manifest impulse control disorders, precipitating behaviors encompassing compulsive gambling, shopping, or excessive eating. The potential emergence of hallucinations, delusions, and other psychiatric manifestations is another facet, particularly discernible at higher dosages [63]. The inception of dopamine agonist therapy might induce nausea and vomiting, although these manifestations tend to ameliorate throughout treatment. An additional consideration encompasses orthostatic hypotension, wherein a precipitous decrease in blood pressure upon standing may lead to feelings of dizziness [64, 65].

Furthermore, the administration of dopamine agonists can perturb sleep architecture, inciting disturbances such as insomnia and diurnal somnolence. The decision surrounding the selection of a specific dopamine agonist and the attendant treatment strategy warrants individualized tailoring contingent on diverse determinants, including chronological age, gravity of symptomatology, and the proclivity for potential side effects. Complemented by regular monitoring and open dialogue with healthcare practitioners, sustained vigilance is incumbent to ascertain that the chosen dopamine agonist achieves its therapeutic objective by efficaciously mitigating symptoms while mitigating the incidence of untoward effects. Adjustments to the therapeutic regimen necessitated by the gradual progression of the disease and evolving patient requirements further underscore the dynamic and personalized nature of PD management [66, 67].

### 4.3 Monoamine Oxidase-B (MAO-B) Inhibitors

Monoamine oxidase type B (MAO-B) inhibitors represent a pharmacotherapeutic cohort extensively employed in treating PD. Their pivotal role resides in ameliorating motor symptoms, at which point they engender their modulatory effects through targeted interaction with the enzyme MAO-B in the cerebral milieu. This enzyme, integral to the catabolism of dopamine, assumes pertinence in the context of PD due to the conspicuous diminution of dopamine resulting from the degeneration of dopamine-producing neurons [68]. Functionally, MAO-B inhibitors are orchestrated to impede the catalytic activity of the MAO-B enzyme. This intervention exerts a restraining influence on the degradation of dopamine, thus translating into augmented levels of dopamine within the cerebral domain. The increase in dopamine concentration heralds symptomatic relief, specifically concerning the motor impairments characteristic of PD, such as tremors, rigidity, and bradykinesia [68]. Two cardinal exemplars within the echelons of MAO-B inhibitors are selegiline and rasagiline. Selegiline, characterized as a selective and irreversible inhibitor of MAO-B, marshals its therapeutic influence by bolstering dopamine concentrations *via* the inhibition of MAO-B-mediated dopamine breakdown. Besides its symptomatic benefits, selegiline has been posited to harbor neuroprotective attributes, potentially engendering retardation in the disease's progressive trajectory. As an adjuvant to levodopa, selegiline is enlisted to assuage motor fluctuations and optimize therapeutic efficacy [69, 70]. Rasagiline, akin to its selegiline counterpart, similarly counteracts the enzymatic breakdown of dopamine by MAO-B. In tandem with levodopa, rasagiline's overarching objective is improving symptom control and mitigating motor fluctuations. The clinical utility of MAO-B inhibitors assumes a multifaceted demeanor. Often incorporated as an adjunct therapy with other pharmacological agents like levodopa, their presence augments the therapeutic potency, particularly in managing motor fluctuations. The prolongation of dopamine availability, achieved through their pharmacological action, serves to temper the variability observed in motor response. Additionally, MAO-B inhibitors may assume the mantle of primary interventions in the nascent stages of PD, especially within cohorts wherein symptomatology remains relatively muted. In such instances, these agents offer symptomatic relief and effectuate a postponement in initiating more potent medications, such as levodopa [71, 72].

Notwithstanding their commendable tolerability, MAO-B inhibitors are not devoid of potential challenges. Particularly germane to selegiline is its propensity to interact with tyramine-rich foods and beverages, thereby affording the risk of precipitating a hypertensive crisis. Adherence to stringent dietary constraints assumes paramount significance for patients under selegiline therapy. Moreover, interactions with other pharmaceutical entities, particularly anti-depressants and select pain medications, entail the potential to evoke serotonin syndrome, a constellation of symptoms bearing clinical significance. MAO-B inhibitors constitute an integral therapeutic cog in PD management. Their strategic targeting of MAO-B underscores their capacity to modify the neurotransmitter milieu, thereby furnishing symptomatic alleviation within the motor domain. While acclaimed for their therapeutic efficacy, vigilance is warranted to mitigate potential interactions and ensure the therapeutic endeavor is conducted within a comprehensive, patient-centric framework [69].

### 4.4 Anticholinergics

Anticholinergic agents, exemplified by compounds like trihexyphenidyl and benztropine, exert their pharmacological action through the antagonism of acetylcholine at muscarinic receptors localized postsynaptically to striatal interneurons. This class of drugs is primarily harnessed for the mitigation of tremor manifestations and does not confer any ameliorative effect upon bradykinesia. The antagonistic action upon acetylcholine, however, engenders a spectrum of untoward effects, encompassing cognitive impairment, confusional states, hallucinatory phenomena, visual disturbances, xerostomia (dry mouth), gastrointestinal dysregulation in the form of constipation, and urinary retention. These pronounced side effects significantly curtail the therapeutic utility of anticholinergic agents within the context of Parkinson's disease management.

### 4.5 Antiglutamatergics

Glutamate stands as the foremost neurotransmitter responsible for mediating excitatory synaptic transmissions and plays a fundamental role in maintaining the brain's physiological functions. Amantadine, originally developed as an antiviral medication for influenza, has emerged as the primary therapeutic agent for addressing levodopa-induced dyskinesia in the context of Parkinson's disease treatment. Beyond its recognized role as an antagonist of glutamate/NMDA receptors, which likely contributes to its efficacy, amantadine exhibits a multifaceted pharmacological profile. One facet of amantadine's action is its purported capacity to stimulate the release of endogenous dopamine stores. By doing so, it augments dopamine levels in the brain, which can help alleviate the motor symptoms associated with Parkinson's disease and the dyskinesias that can arise as a side effect of levodopa therapy. Additionally, amantadine has been implicated in inhibiting the reuptake of synaptic dopamine, thereby enhancing the availability of dopamine in the synaptic cleft and further improving motor function.

Moreover, amantadine possesses anticholinergic properties, meaning it antagonizes the effects of acetylcholine, a neurotransmitter responsible for transmitting signals within the nervous system. This action can lead to various side effects such as dry mouth, constipation, and urinary retention, which are important considerations in its clinical use. The extended-release formulation of amantadine, known as ADS-5102 or Amantadine ER, has been found to be particularly effective. Administered before bedtime, it provides sustained drug release, which leads to improved control over both dyskinesia and motor fluctuations associated with Parkinson's disease. Clinical trials have demonstrated its ability to significantly reduce the mean “off” time (periods when medication is less effective) and increase the mean “on” time without troublesome dyskinesia in patients. Despite its therapeutic benefits, amantadine is associated with certain side effects. Visual hallucinations, peripheral edema (swelling of the extremities), and dizziness are among the most frequently reported adverse effects. These side effects should be monitored closely in patients receiving amantadine therapy. Amantadine ER is available in capsule form with strengths of 68.5 mg and 137 mg. In contrast, another formulation called Osmolex ER is available as tablets with strengths of 129 mg, 193 mg, and 258 mg. Osmolex ER differs from Amantadine ER in its pharmacokinetics, as it delivers amantadine gradually throughout the day. When administered in the morning, it achieves peak plasma concentrations during the waking hours, with lower levels during the nighttime period.

Importantly, amantadine is contraindicated in patients with renal impairment, as its elimination from the body is primarily through the renal route. Dose adjustments or alternative treatments may be necessary for individuals with impaired kidney function to avoid potential toxicity. Amantadine, with its multifaceted mechanisms of action and extended-release formulations, has proven to be a valuable tool in the management of levodopa-induced dyskinesia in Parkinson's disease. However, its use must be carefully monitored, considering both its therapeutic benefits and potential side effects, especially in patients with renal impairment.

## 5 NON-MOTOR SYMPTOMS AND NEUROPHARMACOLOGICAL INTERVENTIONS

**Fig. (3):**
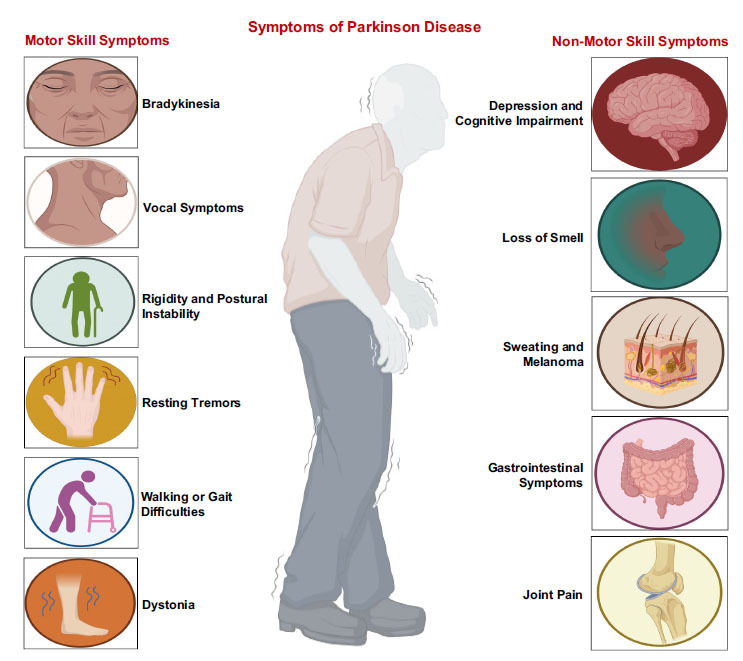
Motor and Non-motor symptoms of Parkinson’s disease.

PD is a neurodegenerative disorder primarily known for its hallmark motor symptoms, including bradykinesia, tremor, rigidity, and postural instability. However, in addition to these motor manifestations, PD is characterized by a wide range of non-motor symptoms (NMS) that significantly impact patients' quality of life and often precede the onset of motor symptoms. This review aims to provide a comprehensive overview of the common non-motor symptoms observed in PD, focusing on cognitive impairment, depression, and psychosis [73] (Fig. **3**).

Cognitive impairment is increasingly recognized as a significant aspect of PD, affecting many patients. It encompasses a spectrum of deficits ranging from mild cognitive impairment (MCI) to full-blown dementia. The cognitive profile in PD is heterogeneous, with impairments in different cognitive domains, including attention, executive function, visuospatial skills, memory, and language. Efforts to address cognitive impairment in PD are centered on modifying neurotransmitter systems involved in cognitive function, particularly cholinergic and glutamatergic pathways. Acetylcholine, a neurotransmitter implicated in attention, memory, and executive function, is significantly depleted in PD. Acetylcholinesterase inhibitors (AChEIs), such as donepezil, rivastigmine, and galantamine, have shown promise in ameliorating cognitive deficits by increasing available acetylcholine [74-76].

Additionally, targeting the glutamatergic system has gained attention. Memantine, an N-methyl-D-aspartate (NMDA) receptor antagonist, is being explored for its potential to regulate glutamate signaling and improve cognitive function. Clinical trials evaluating memantine's efficacy in PD-associated cognitive impairment have yielded mixed results, highlighting the need for further research and optimization of dosing strategies. Deficits in attention and executive function are prominent in PD. Patients may experience reduced concentration ability, increased distractibility, and difficulty switching between tasks. Executive dysfunction includes planning, decision-making, problem-solving, and cognitive flexibility impairments. These cognitive deficits can lead to difficulties in daily activities and contribute to functional disability. Visuospatial impairments involve difficulties in perceiving and interpreting spatial relationships [77]. Patients need help recognizing objects, judging distances, and navigating unfamiliar environments. Visuospatial deficits can contribute to balance and gait disturbances, increasing the risk of falls. Memory deficits in PD primarily affect episodic memory, which involves the ability to remember specific events or experiences. Patients may experience difficulties recalling recent events, learning new information, and retrieving memories. This impairment is thought to result from dysfunction in the medial temporal lobe structures. Reduced verbal fluency, word-finding difficulties, and impaired complex syntax comprehension characterize PD's language deficits. These impairments can lead to communication challenges, affecting social interactions and overall quality of life [78, 79].

Depression is one of the most prevalent non-motor symptoms in PD, occurring in up to 50% of patients at some point during the disease course. It is characterized by persistent low mood, anhedonia, and a range of physical and cognitive symptoms. The etiology of depression in PD is multifactorial, involving neurobiological, psychological, and social factors. Emerging neuropharmacologic strategies for depression in PD focus on targeting multiple neurotransmitter systems implicated in mood regulation, including the serotonergic, noradrenergic, and dopaminergic pathways. Selective serotonin reuptake inhibitors (SSRIs) and serotonin-norepinephrine reuptake inhibitors (SNRIs) are commonly prescribed for treating depression in PD [80, 81]. These drugs enhance the availability of serotonin and norepinephrine in the synaptic cleft, promoting mood stabilization.

Among SSRIs, sertraline has shown efficacy in treating depression while minimizing the risk of interactions with anti-Parkinsonian medications. Dopamine agonists, which primarily target the dopaminergic system, have shown potential in alleviating depression due to their impact on both motor and non-motor symptoms. Pramipexole, a dopamine agonist, has demonstrated anti-depressant effects in PD patients, likely through its actions on D2/D3 receptors and the reward pathway. Novel approaches are also being explored. For instance, ketamine, an NMDA receptor antagonist, has shown rapid and robust anti-depressant effects in treatment-resistant depression. Clinical trials investigating ketamine's potential in PD-related depression are underway, shedding light on its role in managing this complex symptom. Dopaminergic dysfunction is implicated in the pathophysiology of both motor and non-motor symptoms in PD, including depression. Degeneration of dopaminergic neurons in the substantia nigra contributes to dopamine depletion, which plays a role in regulating mood [82, 83]. Deregulation of other neurotransmitter systems, such as serotonin and norepinephrine, also contributes to depressive symptoms. Living with a chronic and progressive disease like PD can lead to psychological distress, contributing to depression. Patients may experience frustration, grief over lost abilities, and anxiety about the future. The physical limitations imposed by motor symptoms can lead to feelings of helplessness and dependency, further exacerbating depressive symptoms. Psychosis in PD refers to a spectrum of symptoms, including hallucinations and delusions. Although the prevalence varies, up to 60% of PD patients may experience psychotic symptoms during the disease course. Psychosis significantly impacts patient and caregiver well-being, often leading to increased burden and reduced quality of life. Managing psychosis in PD involves a delicate balance between addressing symptoms and minimizing exacerbation of motor dysfunction [84, 85]. Emerging strategies encompassing both pharmacological and non-pharmacological interventions are being explored. Pimavanserin, a selective serotonin inverse agonist/antagonist at the 5-HT2A receptor, is a groundbreaking drug approved for treating PD psychosis. Unlike traditional anti-psychotics, pimavanserin does not worsen motor symptoms due to its minimal affinity for dopamine receptors. Clinical trials have demonstrated its efficacy in reducing hallucinations and delusions without compromising motor function. Clozapine, an atypical anti-psychotic, is being investigated for its potential in PD psychosis. Despite its potential motor side effects, its superior efficacy in managing psychosis refractory to other treatments justifies its exploration. Tailored dosing and careful monitoring can mitigate motor concerns. Non-pharmacological interventions, such as cognitive-behavioral therapy and psychoeducation, are emerging as adjunctive strategies for managing PD psychosis [86, 87]. These interventions address cognitive distortions and maladaptive beliefs, enhancing patients' coping mechanisms and overall well-being. Visual hallucinations are the most common type of psychotic symptom in PD. Patients may perceive objects, people, or animals that are not present. These hallucinations often involve benign content but can sometimes become distressing or disruptive. The underlying neural mechanisms involve alterations in visual processing pathways and deregulation of neurotransmitter systems. Delusions in PD are typically paranoid or persecutory. Patients may develop irrational beliefs that they are being spied on, followed, or plotted. Delusions can contribute to caregiver stress and pose challenges for clinical management. The neurobiological basis of psychosis in PD involves a complex interplay of dopamine deregulation, cholinergic dysfunction, and alterations in neural circuits. Overstimulation of dopamine receptors, particularly the D2 receptors, is implicated in developing hallucinations and delusions. Additionally, cholinergic deficits in the basal forebrain contribute to cognitive impairments and may play a role in psychosis [88, 89].

PD is a multifaceted disorder characterized by motor symptoms and various non-motor symptoms. Cognitive impairment, depression, and psychosis are prominent NMS that significantly impact patients' quality of life. The heterogeneity of these symptoms underscores the complex underlying pathophysiological mechanisms involving dopaminergic and other neurotransmitter systems and neural circuitry alterations. Early recognition, accurate diagnosis, and tailored management of these non-motor symptoms are crucial for optimizing the overall care and well-being of individuals with PD. While emerging neuropharmacologic strategies hold promise in addressing non-motor symptoms in PD, several challenges must be acknowledged. Interactions with existing anti-Parkinsonian medications, potential side effects, and patient-specific responses necessitate individualized treatment approaches [4, 90].

Furthermore, long-term efficacy and safety data are crucial to establish the sustained benefits of these interventions. Future directions in this field involve a deeper understanding of the underlying neurobiology of non-motor symptoms. Identifying biomarkers associated with cognitive impairment, depression, and psychosis could facilitate early diagnosis and targeted interventions. Incorporating genetic, neuroimaging, clinical data, and personalized medicine approaches may enable tailored treatment strategies based on patients' unique profiles.

## 6 ETHNOPHARMACOLOGICAL APPROACHES IN PD

PD, a progressively debilitating neurodegenerative disorder, presents a complex array of motor and non-motor symptoms that significantly impact patients' quality of life. While conventional treatments alleviate some of these symptoms, developing effective disease-modifying interventions remains challenging. In this context, ethnopharmacologic approaches, which integrate traditional knowledge with modern scientific methodologies, have emerged as a promising avenue for identifying and validating potential interventions for PD. Ethnopharmacology, a multidisciplinary field amalgamating insights from anthropology, botany, pharmacology, and chemistry, involves exploring indigenous medical practices to uncover bioactive compounds and formulations with therapeutic potential [4]. This approach acknowledges the profound connection between culture, nature, and health while leveraging traditional medicine's empirical wisdom to inform contemporary research. Traditional medicine systems are intrinsic to diverse cultures across the globe, embodying accumulated knowledge and practices passed down through generations. These systems, such as Ayurveda, Traditional Chinese Medicine (TCM), Indigenous Medicine, and African Traditional Medicine, encompass holistic approaches considering the interconnectedness of the body, mind, and environment [91, 92]. The wisdom encapsulated within these systems can provide valuable insights into PD's etiology, progression, and management. The relevance of traditional medicine systems to modern drug discovery is exemplified by their historical use of plant-based remedies to manage a range of ailments, including neurological disorders. These remedies, often based on observations of medicinal plants' effects on symptoms resembling those of PD, have withstood the test of time. As modern science unravels these natural substances' chemical complexity and mechanisms, traditional medicine systems serve as a reservoir of hypotheses for further investigation. Plant-based remedies constitute a cornerstone of traditional medicine systems and have been utilized for centuries to address neurological disorders [93]. The historical utilization of herbs such as *Mucuna pruriens*, Withania *somnifera*, and Ginkgo *biloba* in various cultures underscores the enduring recognition of their potential neurological benefits. These plants are replete with compounds such as alkaloids, flavonoids, polyphenols, and terpenoids that exhibit diverse bioactivities, including anti-oxidative, anti-inflammatory, and neuroprotective properties [94, 95].

The historical use of plant-based remedies for conditions reminiscent of PD symptoms attests to the sustained relevance of these natural substances in addressing neurological disorders. For instance, *Mucuna pruriens*, commonly known as Velvet bean, is historically associated with managing movement disorders. The presence of L-DOPA in *Mucuna pruriens* seeds, a precursor to dopamine, underscores the botanical basis for traditional remedies that impact neurotransmitter imbalances in PD. Many plant-derived bioactive compounds possess neuroprotective attributes that align with PD's pathophysiological hallmarks. Flavonoids, for instance, exhibit anti-oxidative and anti-inflammatory properties that counteract oxidative stress and neuroinflammation, critical contributors to PD progression [96, 97]. Curcumin, derived from turmeric, has shown potential as a neuroprotective agent due to its pleiotropic effects, including anti-amyloid and anti-inflammatory actions. The convergence of traditional concepts and modern neurobiology offers a dualistic perspective on PD etiology and treatment. Traditional medicine systems often describe disease processes through energetic imbalances, humoral theory, and organ systems. Though differing in language and framework, these concepts resonate with modern neurobiology's understanding of neural circuitry, neurotransmitter imbalances, and neuroinflammation. While ethnopharmacological approaches hold promise, they entail challenges in translating traditional remedies into modern therapeutics. Issues related to standardization, quality control, and validation of conventional medicine for clinical application warrant rigorous investigation. Ethical considerations also come to the fore, necessitating the equitable engagement of local communities and respect for their traditional knowledge. Integrating traditional medicine systems with modern drug discovery represents a confluence of traditions and innovation [98, 99]. As contemporary research methodologies, including *in vitro* assays, animal models, and clinical trials, validate the efficacy and safety of ethnopharmacologic interventions, the divide between traditional wisdom and evidence-based medicine diminishes. This convergence offers a holistic approach to addressing the multifaceted challenges of PD [100, 101].

Ethnopharmacologic approaches offer a bridge between the rich tapestry of traditional medicine systems and the rigorous methods of modern drug discovery. The historical utilization of plant-based remedies across cultures, informed by empirical observation and accumulated knowledge, aligns with contemporary research's goal to uncover effective interventions for PD. By acknowledging the interaction between tradition and modernity, ethnopharmacology holds promise in shaping the future of PD therapeutics through a harmonious synthesis of ancient wisdom and cutting-edge science [102] (Table **1**).

## 7 NATURAL COMPOUNDS WITH ANTI-PARKINSONIAN PROPERTIES

**Fig. (4):**
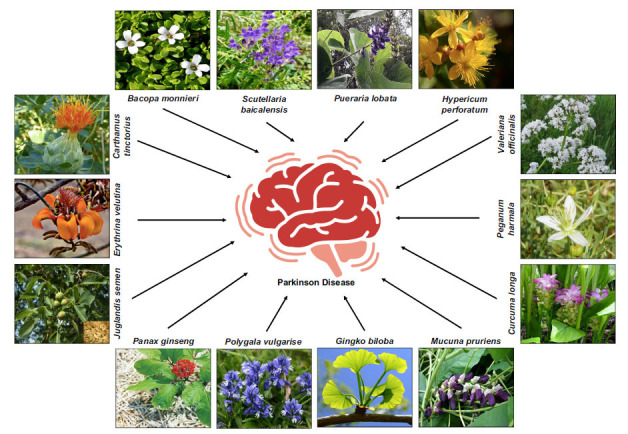
Natural compounds as potential interventions for Parkinson’s diseae.

PD is a complex neurodegenerative disorder characterized by the progressive degeneration of dopaminergic neurons, leading to motor and non-motor symptoms. Conventional therapies often address the motor symptoms but do not provide a comprehensive solution for disease modification. Exploring natural compounds as potential interventions for PD has gained traction due to their diverse bioactive properties. This review delves into specific natural compounds, including curcumin, resveratrol, and flavonoids, discussing their neuroprotective effects, anti-inflammatory actions, and impact on oxidative stress. Furthermore, the potential mechanisms underlying these compounds' benefits are elucidated [4] (Fig. **4**).

### 7.1 Curcumin

Curcumin, a polyphenolic compound sourced from the rhizomes of *Curcuma longa*, has attracted considerable scientific interest owing to its versatile neuroprotective effects, particularly in the context of PD. This bioactive compound has demonstrated a capacity to modulate diverse molecular pathways intricately associated with the pathogenesis of PD, encompassing neuroinflammation, oxidative stress, and protein misfolding. One pivotal facet of curcumin's neuroprotective prowess lies in its potent ability to mitigate neuroinflammation, a hallmark feature of PD. Under its pronounced anti-inflammatory properties, curcumin orchestrates the attenuation of pro-inflammatory mediators that drive neuroinflammatory responses. Among these are cytokines such as tumor necrosis factor-alpha (TNF-α) and interleukin-1β (IL-1β), which curcumin has demonstrated efficacy in suppressing. This suppression, in turn, impedes the activation of microglia and astrocytes, the central nervous system's resident immune cells, thereby curbing the propagation of neuroinflammation cascades implicated in neuronal degeneration. Curcumin's role as a robust anti-oxidant further underpins its neuroprotective effects [103, 104].

PD pathophysiology is underpinned by oxidative stress, arising from an imbalance between reactive oxygen species (ROS) production and the endogenous antioxidant defense system. Curcumin bolsters the cellular anti-oxidant defense repertoire through its capacity to neutralize ROS and reactive nitrogen species (RNS). Furthermore, this polyphenolic compound augments the activity of key anti-oxidant enzymes, including superoxide dismutase (SOD) and catalase, pivotal players in ROS scavenging. This intricate interplay between curcumin and cellular anti-oxidants synergistically alleviates oxidative stress, reducing the pro-oxidative burden contributing to neurodegeneration. Protein misfolding and aggregation, typified by α-synuclein, are prominent features of PD pathogenesis. Curcumin's ability to influence protein folding dynamics is vital to its neuroprotective effects [17, 105]. By binding to α-synuclein aggregates, curcumin interferes with their assembly, impeding the formation of toxic oligomers and fibrils. This molecular interaction mitigates the detrimental impact of protein misfolding but also assists in promoting protein clearance mechanisms, such as autophagy and proteasomal degradation. As a result, curcumin's actions reduce the accumulation of aberrant protein aggregates known to exert neurotoxic effects. The multifaceted neuroprotective effects of curcumin stem from its capacity to interact with an array of cellular signaling pathways. One such pathway is the nuclear factor erythroid 2-related factor 2 (Nrf2) pathway, which is pivotal in regulating anti-oxidant response elements. Curcumin's modulation of Nrf2 enhances the transcription of anti-oxidant enzymes, bolstering cellular defense mechanisms against oxidative stress. Additionally, curcumin inhibits nuclear factor-kappa B (NF-κB), a master regulator of inflammation. Doing so curtails the expression of pro-inflammatory genes, contributing to the attenuation of neuroinflammation [106, 107].

Curcumin's multifaceted neuroprotective effects render it a promising candidate for addressing the intricate pathophysiological underpinnings of PD. Its capacity to modulate neuroinflammation, alleviate oxidative stress, and attenuate protein misfolding showcases a comprehensive approach that intersects with key molecular cascades implicated in PD's etiology. By engaging with cellular signaling pathways and orchestrating a multifunctional response, curcumin exemplifies a bioactive compound with potential therapeutic utility in managing PD.

### 7.2 Resveratrol

Resveratrol, a polyphenolic compound inherent to grapes and red wine, has garnered substantial scientific attention due to its emerging potential for conferring neuroprotection in the context of PD. Emanating from its versatile nature, resveratrol engenders a spectrum of modulatory effects across intricate molecular pathways, encompassing mitochondrial function, oxidative stress, and cellular survival mechanisms. A hallmark feature of resveratrol's neuroprotective profile is its influence on mitochondrial function, a critical factor in PD pathogenesis. Resveratrol orchestrates a multifaceted response by interacting with sirtuin 1 (SIRT1), a NAD^+^-dependent deacetylase implicated in various cellular processes. The activation of SIRT1 by resveratrol potentiates the deacetylation of peroxisome proliferator-activated receptor-gamma coactivator-1 alpha (PGC-1α), a master regulator of mitochondrial biogenesis [108, 109].

Consequently, this modulation enhances gene expression in mitochondrial function and biogenesis. The intricate orchestration of these events culminates in improved mitochondrial energy metabolism, fostering cellular resilience against pathological insults. Resveratrol's anti-inflammatory properties are integral to its neuroprotective profile in PD. This polyphenol, by its capacity to inhibit the expression of inflammatory mediators, intervenes in the signaling cascades that drive neuroinflammation. Notably, resveratrol hampers the induction of inducible nitric oxide synthase (iNOS) and cyclooxygenase-2 (COX-2), key enzymes in generating pro-inflammatory species. This attenuation of inflammatory mediators extends to microglial activation, curbing their propensity to perpetuate the inflammatory milieu [110, 111].

Consequently, resveratrol's anti-inflammatory actions are instrumental in mitigating neuroinflammation, a pivotal contributor to PD pathogenesis. Oxidative stress, a prominent feature of PD, is a critical target of resveratrol's neuroprotective endeavors. Resveratrol's anti-oxidative properties are manifold, encompassing both direct and indirect mechanisms. This compound exerts a direct anti-oxidant effect by scavenging reactive oxygen species (ROS) and reactive nitrogen species (RNS), thereby abating their potential to inflict cellular damage. Additionally, resveratrol augments the endogenous anti-oxidant defense mechanisms, amplifying the activity of antioxidant enzymes like superoxide dismutase (SOD) and catalase [112, 113]. Of paramount importance is resveratrol's activation of SIRT1, which subsequently fosters mitochondrial biogenesis and efficiency. Resveratrol indirectly diminishes ROS production by bolstering mitochondrial function, further ameliorating oxidative stress. Resveratrol's salutary effects in PD are intricately linked to its activation of SIRT1 and subsequent consequences. The orchestration of mitochondrial biogenesis through the modulation of PGC-1α, a pivotal regulator of mitochondrial function, enhances energy metabolism and diminishes oxidative stress. Resveratrol's augmentation of SIRT1 also exerts a transformative influence on the autophagy-lysosome pathway, a cellular process vital for removing damaged organelles and protein aggregates. This facilitation of protein clearance mechanisms reduces protein misfolding and subsequent neurotoxicity, fundamental hallmarks of PD pathology. In sum, resveratrol emerges as a multifaceted candidate for neuroprotection in PD under its modulation of intricate molecular pathways [111, 114]. Through its facilitation of mitochondrial biogenesis, anti-inflammatory actions, and impact on oxidative stress, resveratrol presents a comprehensive approach that aligns with the complex etiology of PD. The activation of SIRT1 and the subsequent orchestration of downstream events underscore the compound's capacity to counteract neurodegenerative processes. As our understanding of these mechanisms deepens, resveratrol holds promise as a valuable avenue in the pursuit of innovative therapeutic strategies for PD [115].

### 7.3 Flavonoids

Flavonoids, a diverse class of polyphenolic compounds widely distributed in various plant sources, have garnered scientific attention due to their potential neuroprotective properties, particularly in the context of PD. Within this class, certain flavonoids, notably quercetin and epigallocatechin gallate (EGCG), have emerged as intriguing candidates for mitigating the cellular pathways integral to PD pathogenesis [116, 117]. The neuroprotective potential of flavonoids resides in their capacity to influence cellular pathways intricately involved in PD pathophysiology. This spans neuroinflammation, oxidative stress, and protein misfolding, key facets contributing to neuronal degeneration. Flavonoids exhibit substantial anti-inflammatory attributes, fundamentally rooted in their ability to regulate nuclear factor-kappa B (NF-κB) signaling. This regulatory role underscores their potential to mitigate the production of pro-inflammatory cytokines and chemokines, curbing the inflammatory milieu orchestrated by microglial activation. Through suppressing NF-κB-mediated transcriptional events, flavonoids curtail the perpetuation of neuroinflammation, an essential driver of neurodegenerative processes in PD [118, 119]. A defining hallmark of PD is oxidative stress, driven by an imbalance between reactive oxygen species (ROS) and the cellular antioxidant defense system. Flavonoids emerge as potent anti-oxidants, effectively neutralizing ROS and reactive nitrogen species (RNS). Their mode of action extends to enhancing the activity of pivotal anti-oxidant enzymes such as superoxide dismutase (SOD) and glutathione peroxidase. By reinforcing endogenous anti-oxidant defenses, flavonoids mitigate oxidative damage and bolster cellular resilience against stressors implicated in PD pathogenesis. Flavonoids' neuroprotective effects are attributed to their interactions with multiple signaling pathways, offering a nuanced and comprehensive approach to PD intervention [27, 120].

#### 7.3.1 Quercetin

Quercetin, for instance, engages the nuclear factor erythroid 2-related factor 2 (Nrf2) pathway, which is pivotal in the transcriptional regulation of anti-oxidant and detoxification genes. The activation of Nrf2 by quercetin culminates upregulating a diverse array of genes responsible for bolstering cellular anti-oxidant defenses. This orchestrated response mitigates oxidative stress and promotes cellular detoxification machinery, aiding in the clearance of toxic compounds that contribute to neuronal degeneration [121, 122].

#### 7.3.2 EGCG

Epigallocatechin gallate (EGCG), prominent in green tea, exerts its neuroprotective effects through distinct mechanisms. EGCG's impact on mitochondrial function holds significance, influencing mitochondrial membrane potential and ROS production. By modulating mitochondrial function, EGCG indirectly reduces ROS burden, alleviating oxidative stress. Furthermore, EGCG's interaction with α-synuclein manifests through its capacity to inhibit protein aggregation. This interaction could mitigate the neurotoxic effects associated with protein misfolding [123, 124].

Flavonoids, such as quercetin and EGCG, underscore their potential as neuroprotective agents in the intricate landscape of PD. Through their modulation of cellular pathways encompassing neuroinflammation, oxidative stress, and protein misfolding, flavonoids offer a multi-dimensional approach to counteracting PD's pathophysiology. The intricate mechanisms underlying their benefits, ranging from Nrf2 activation to mitochondrial modulation and protein interaction, reflect their versatility in addressing the multifaceted challenges of PD. As our understanding deepens, flavonoids hold promise as integral components in the quest for innovative therapeutic strategies against PD [118, 125].

## 8 TRADITIONAL HERBAL FORMULATIONS AND THEIR MECHANISMS OF ACTION

The utilization of traditional herbal formulations for managing PD has a rich history spanning diverse cultures. These formulations often harness the collective wisdom of generations, offering insights into novel therapeutic approaches. Two such formulations, *Mucuna pruriens* (Velvet bean) and *Bacopa monnieri*, stand out due to their significant utilization in PD management. Examining their active constituents and potential modes of action provides valuable insights into their therapeutic potential. At the same time, the concept of synergistic effects within multi-ingredient formulations adds a layer of complexity to their efficacy [126, 127].

### 8.1 *Mucuna pruriens* (Velvet bean)

*Mucuna pruriens*, commonly recognized as Velvet bean, occupies a prominent place within diverse traditional medicine systems due to its perceived efficacy in ameliorating symptoms associated with PD. At the heart of its therapeutic potential lies the primary active constituent, L-DOPA (levodopa), which holds immense significance in PD management. This is attributed to its role as a precursor to dopamine, a neurotransmitter whose depletion is a hallmark of PD pathology [96, 128]. Integrating *Mucuna pruriens* into traditional practices aligns harmoniously with the conventional strategy of elevating dopamine levels within the brain. *Mucuna pruriens* is distinguished by its high content of L-DOPA, which constitutes a cornerstone of its therapeutic action. L-DOPA is an essential amino acid precursor to dopamine, the neurotransmitter critically implicated in motor control and emotional regulation. In the context of PD, where dopamine-producing neurons are compromised, providing L-DOPA through *Mucuna pruriens* is strategically aligned to restore dopaminergic function. The therapeutic effects of *Mucuna pruriens* in PD can be attributed to the cascade of events set in motion by converting L-DOPA into dopamine within the brain [96, 129, 130]. This process, facilitated by the enzyme aromatic L-amino acid decarboxylase (AADC), effectively addresses the neurotransmitter deficiency characteristic of PD. The subsequent increase in dopamine levels serves as the foundation for alleviating the motor symptoms that define PD, including tremors, rigidity, and bradykinesia. Remarkably, *Mucuna pruriens*' impact is not confined solely to motor symptoms. The restoration of dopamine levels also extends its beneficial influence to non-motor domains of PD. These encompass mood regulation, cognitive function, and overall quality of life. By enhancing dopaminergic activity in brain regions associated with mood and cognition, *Mucuna pruriens* can potentially ameliorate depression, anxiety, and cognitive impairments often experienced by PD patients [33, 131]. In traditional medicine, *Mucuna pruriens* is often a pivotal component within multi-ingredient formulations. These formulations are meticulously crafted to optimize the therapeutic potential of *Mucuna pruriens* while synergistically engaging with other herbs and compounds. Such formulations frequently incorporate adaptogens, anti-inflammatory agents, and anti-oxidants. By combining *Mucuna pruriens* with adaptogenic herbs, these formulations aim to enhance the body's resilience to stressors, thereby supporting the overall well-being of PD patients. Including anti-inflammatory agents addresses the neuroinflammatory component of PD pathogenesis, which can exacerbate neuronal degeneration. Additionally, anti-oxidants contribute to mitigating oxidative stress, a process implicated in the progression of PD [132, 133].

*Mucuna pruriens* stands as a testament to integrating traditional wisdom into modern approaches to PD management. L-DOPA, coupled with its conversion to dopamine, addresses motor symptoms and extends its reach to mood regulation and cognitive enhancement. Furthermore, incorporating *Mucuna pruriens* within multi-ingredient formulations accentuates its therapeutic impact, offering a holistic approach that encompasses dopamine replenishment, neuroinflammation attenuation, and oxidative stress mitigation. This ancient herbal remedy, grounded in its bioactive constituents and intricate interactions, continues to unveil its potential as a valuable adjunct in managing PD [97].

### 8.2 *Bacopa monnieri* 

*Bacopa monnieri*, an integral facet of Ayurvedic medicine, has emerged as a subject of interest in the context of PD management. This botanical treasure trove harbors active constituents known as bacosides, triterpenoid saponins endowed with neuroprotective attributes. The allure of *Bacopa monnieri* stems from its capacity to traverse a gamut of cellular pathways pertinent to PD pathogenesis, thereby offering a holistic approach to intervention. At the heart of *Bacopa monnieri's* therapeutic prowess lie its bacosides, triterpenoid saponins that encapsulate its neuroprotective potential. These bioactive compounds are a product of nature's chemical craftsmanship, meticulously tailored by evolution to engage with neural intricacies. Bacosides illuminate a multifaceted mechanism of action that converges on the convergence of neurodegeneration in PD. Foremost among their attributes are their anti-oxidant and anti-inflammatory prowess. By engaging in a dance with oxidative stress, bacosides bestow the neurons with bolstered anti-oxidant defenses. This orchestration not only scuttles the perilous cascade of oxidative damage but also contributes to abating the oxidative stress that acts as a catalyst for PD progression [134].

Furthermore, bacosides unfurl their potential as anti-inflammatory agents, engaging in a delicate choreography with neuroinflammation. By restraining the deployment of pro-inflammatory cytokines, bacosides attenuate the neuroinflammatory cacophony orchestrated by microglial activation. This intervention not only eases inflammation-associated neuronal damage but also ensures the mitigation of PD-associated motor and cognitive impairments. *Bacopa monnieri's* impact transcends quelling inflammation and oxidative stress. Its bacosides unveil the attributes of neurotrophic promotion, which fuels neuronal vitality [135]. By activating neurotrophic factors, bacosides kindle the spark of neuronal growth, differentiation, and survival. This expansive embrace of neurotrophic support has the potential to defy the storm of neuronal degeneration, a hallmark of PD pathology. In the tapestry of traditional medicine, *Bacopa monnieri* often finds itself interwoven within multi-ingredient formulations, akin to an artistic collaboration. These formulations epitomize the synergy within nature's pharmacopeia. Integrating *Bacopa monnieri* with herbs housing complementary attributes begets a symphony of therapeutic effects [136, 137]. These multi-ingredient formulations exhibit a harmonious orchestration against the multifaceted tapestry of PD pathogenesis. The presence of anti-inflammatory herbs dampens the neuroinflammatory conflagration, while anti-oxidants further strengthen the resilience of neurons against oxidative onslaught. The convergence of these attributes fashions a comprehensive and nuanced approach that navigates PD's complex etiology [138].

*Bacopa monnieri* emerges as a beacon of hope in PD management. Its bacosides' ability to traverse the landscape of oxidative stress, inflammation, and neuronal vitality establishes a multifaceted therapeutic approach. This natural masterpiece is often brought to life within formulations that harness the symphony of synergy, creating a comprehensive resonance against the symphony of PD pathogenesis. Amidst neural intricacies, *Bacopa monnieri* heralds a harmonious melody of neuroprotection [139].

## 9 TRADITIONAL PHARMACOLOGIC APPROACH IN PARKINSON'S DISEASE

Parkinson's disease (PD) management typically involves a combination of pharmacologic strategies aimed at alleviating symptoms and improving quality of life. These strategies can be broadly categorized into natural management, which includes the use of plant-based remedies, and conventional drug management, which relies on synthetic pharmaceuticals. Natural management of PD involves the use of plant-derived compounds that have shown potential neuroprotective and symptom-relieving properties. Key examples include *Mucuna pruriens*, *Bacopa monnieri*, and curcumin. *Mucuna pruriens* contains high levels of levodopa, a precursor to dopamine, which can cross the blood-brain barrier and supplement the depleted dopamine levels in PD patients. Studies suggest it may have fewer side effects compared to synthetic levodopa. *Bacopa monnieri* is known for its neuroprotective and cognitive-enhancing properties, potentially mitigating cognitive decline and neuroinflammation in PD. Curcumin, the active compound in turmeric, exhibits potent anti-inflammatory and antioxidant properties, potentially protecting dopaminergic neurons from degeneration.

Conventional pharmacologic treatments for PD primarily involve synthetic drugs designed to either replace or mimic dopamine or to modulate other neurotransmitter systems to manage symptoms. Levodopa is the cornerstone of PD treatment and is converted to dopamine in the brain, providing symptomatic relief. It is often combined with carbidopa to prevent peripheral metabolism and enhance central availability. Dopamine agonists, such as pramipexole and ropinirole, directly stimulate dopamine receptors and are used as monotherapy in early PD or as adjuncts to levodopa in advanced stages to smooth motor fluctuations. MAO-B inhibitors, including selegiline and rasagiline, inhibit the enzyme monoamine oxidase B, which breaks down dopamine, thereby increasing its availability in the brain [69]. While both natural and conventional drug management strategies aim to address the dopaminergic deficiency in PD, their mechanisms and effects vary significantly. Natural treatments derive from plant sources and often contain a mixture of bioactive compounds, which may provide a broader range of therapeutic effects and potentially fewer side effects. In contrast, conventional drugs are synthetic and typically designed to target specific pathways or receptors. Natural remedies like *Mucuna pruriens* are reported to have fewer side effects compared to synthetic levodopa, although comprehensive clinical trials are needed to confirm these findings. Conventional drugs, while effective, often come with a higher risk of side effects, such as dyskinesia from long-term levodopa use or impulse control disorders from dopamine agonists. Natural management tends to take a more holistic approach, potentially addressing multiple aspects of PD pathology, including neuroinflammation and oxidative stress. In contrast, conventional pharmacotherapy is more targeted, focusing on specific symptoms or pathways, which may necessitate polypharmacy to address the full spectrum of PD symptoms. Conventional drugs have undergone extensive clinical testing and are widely accepted in medical practice. In contrast, while there is growing evidence supporting the efficacy of certain natural compounds, they are not as rigorously studied or universally adopted in clinical practice.

## 10 SYNERGISTIC EFFECTS OF MULTI-INGREDIENT FORMULATIONS

Traditional herbal formulations, informed by centuries of empirical wisdom, frequently espouse the principle that combining multiple ingredients culminates in therapeutic synergism, transcending the sum of individual components. This enigmatic yet intuitive phenomenon is recognized as a cornerstone of traditional medicine's efficacy. In the context of PD, this approach is manifest in formulations encompassing *Mucuna pruriens* and *Bacopa monnieri*, where the interplay of diverse components orchestrates a symphony of effects distinctly tailored to the multifaceted landscape of PD pathophysiology [140, 141]. Synergy encapsulates the intricate interplay of compounds, engendering effects that surpass the predictive sum of their actions. In traditional medicine systems, this concept is harnessed to craft formulations that synchronize with the body's complexities, offering a holistic approach. This harmonious orchestration resonates poignantly within neurodegenerative disorders like PD, characterized by the convergence of diverse pathological factors. Within the milieu of PD, formulations that incorporate *Mucuna pruriens* and *Bacopa monnieri* epitomize the power of synergy. These formulations are emblematic of holistic approaches that engage with PD's intricate pathophysiology at multiple junctures rather than employing a narrow, mono-dimensional strategy. The essence of synergy lies in its capacity to address multiple facets of PD pathophysiology simultaneously, enhancing the overall therapeutic effect. For example, formulations encompassing *Mucuna pruriens* not only furnish L-DOPA for dopamine replenishment but also incorporate adaptogenic herbs to bolster the body's stress response, anti-inflammatory agents to curtail neuroinflammation, and anti-oxidants to counteract oxidative stress. This orchestrated symphony attends to dopamine deficiency while concurrently alleviating inflammation and oxidative damage [142, 143].

Similarly, formulations integrating *Bacopa monnieri* intertwine its neuroprotective attributes with those of complementary herbs. Anti-inflammatory herbs curb neuroinflammatory cascades, anti-oxidants mitigate oxidative stress, and adaptogens enhance stress resilience. This multifaceted engagement safeguards neurons against an intricate tapestry of PD-induced challenges [144].

The essence of synergy resides in its capacity to amplify therapeutic effects by converging upon multiple targets, simultaneously abating several pathological processes, where the convergence of factors necessitates a comprehensive intervention. By coalescing the potency of diverse compounds, these formulations transcend the boundaries of individual contributions, ushering forth an enhanced and nuanced therapeutic outcome. The synergy witnessed within multi-ingredient formulations embracing *Mucuna pruriens*, and *Bacopa monnieri* reflects the intricate tapestry of nature's pharmacopeia. This harmonization of compounds harmoniously resonates with the complexity of PD's pathophysiology, embodying the essence of traditional medicine's wisdom. The efficacy of these formulations illuminates the path toward a holistic, multi-dimensional approach to addressing the intricate challenges of PD [145, 146]. The synergistic interactions manifesting within traditional herbal formulations, encompassing diverse components like *Mucuna pruriens* and *Bacopa monnieri*, exemplify the holistic ethos of traditional medicine. These formulations strategically target multiple facets of PD pathophysiology, harmonizing an array of therapeutic actions into a unified and enhanced response. Amidst the intricate symphony of PD's complexities, the concept of synergy emerges as a guiding principle, paving the way for nuanced and comprehensive interventions [147, 148].

## 11 ETHNOBOTANICAL INSIGHTS AND CULTURAL CONSIDERATIONS

Ethnopharmacology is intrinsically interwoven with cultural practices and traditional knowledge, offering a profound avenue for unraveling indigenous treatments for complex disorders such as PD. This paradigm merges science and cultural heritage, tapping into the rich tapestry of ancestral wisdom and botanical resources that Indigenous communities have cultivated over generations. In the context of PD, ethnobotanical studies wield the power to unearth therapeutic treasures deeply entrenched in the traditions of diverse cultures while concurrently spotlighting the importance of cultural sensitivity, knowledge preservation, and ethical considerations [149, 150]. Ethnobotanical studies serve as a bridge between traditional medicine and contemporary scientific inquiry. These studies delve into the repositories of local knowledge, unearthing the botanical riches and practices that have stood the test of time. In the realm of PD, where conventional interventions may offer incomplete solutions, ethnobotanical research unveils an array of treatments cultivated within the cultural and ecological contexts of different communities [151].

Indigenous communities often possess a nuanced understanding of local plants, their applications, and their therapeutic potential. This deep-rooted wisdom, handed down through generations, offers an invaluable reservoir of knowledge. Ethnopharmacological exploration unveils hidden gems like *Mucuna pruriens* and *Bacopa monnieri*, which have demonstrated significant promise in PD management. These discoveries highlight the unique contributions of indigenous treatments that may complement or even enhance mainstream therapeutic approaches [152, 153]. Engagement with indigenous communities necessitates a foundation of cultural sensitivity and respect. These communities hold intimate relationships with their environment, and their healing practices are often intertwined with sacred rituals, customs, and beliefs. Researchers must approach these collaborations humbly, acknowledging cultural preservation's profound importance. Cultural sensitivity extends beyond respectful engagement to embracing local terminologies, rituals, and perspectives to foster mutual understanding and co-learning. Ethnopharmacological research presents the dual responsibility of uncovering ancient wisdom and preserving it for future generations. Documenting traditional knowledge safeguards the intangible heritage embedded within these communities. Collaborative efforts between researchers and local practitioners can create comprehensive databases that preserve indigenous plant use, preparation methods, and healing rituals. Such endeavors are crucial to ensuring the continuity of this invaluable repository of knowledge [154].

Collaborations with indigenous communities demand ethical considerations that extend beyond traditional research protocols. Informed consent, community participation, and equitable benefit-sharing are paramount. Researchers must ensure that these collaborations result in mutually beneficial outcomes, including empowering indigenous practitioners and communities. Respect for intellectual property rights and protection against biopiracy further underpin the ethical framework. In essence, ethnopharmacology offers a pathway to amplify the therapeutic landscape for PD through the marriage of ancestral knowledge and modern scientific rigor. This journey requires an unwavering commitment to cultural sensitivity, ethical responsibility, and knowledge preservation. As researchers navigate the intricate tapestry of ethnopharmacology, they weave a narrative that honors the wisdom of the past while embracing the potential of the future—a narrative that both elevates the scientific pursuit and celebrates the profound cultural contributions to healing practices [155].

## 12 CHALLENGES AND OPPORTUNITIES

Exploring ethnopharmacologic interventions against PD offers a tantalizing prospect of uncovering novel therapeutic avenues rooted in traditional knowledge. However, this pursuit is challenging, as translating ancient wisdom into evidence-based treatments requires navigating intricate terrains. Issues surrounding standardization, quality control, knowledge translation, and ethical considerations such as biopiracy and benefit-sharing agreements emerge as significant hurdles in the journey from indigenous practices to contemporary healthcare. One of the foremost challenges in integrating ethnopharmacologic interventions into modern healthcare systems is the standardization of herbal remedies. Indigenous treatments often encompass complex mixtures of plants, making it essential to define consistent parameters for identification, collection, processing, and dosage [156]. Inconsistencies in preparation and administration can lead to variations in therapeutic outcomes, hindering reproducibility and comparability. Developing standardized protocols and quality control measures is pivotal to ensuring safety and efficacy and bridging the gap between traditional practices and evidence-based medicine. Translating traditional knowledge into evidence-based treatments is a delicate process that demands meticulous attention. Indigenous healing practices often rely on holistic approaches that may not neatly align with reductionist scientific methodologies. Deciphering the underlying mechanisms of action and establishing causality poses challenges, as does the integration of traditional and modern medical paradigms. Rigorous scientific investigation is required to validate the therapeutic claims while respecting the holistic context within which these practices originated [157]. The potential for biopiracy, the unauthorized commercial exploitation of traditional knowledge and genetic resources, casts a shadow over ethnopharmacologic research. As indigenous treatments gain recognition, there is a risk that proprietary interests may exploit these resources without equitable benefit-sharing with the originating communities. To safeguard against such exploitation, mechanisms such as the Nagoya Protocol have been established to ensure that benefits are shared with local communities fairly and justly. Researchers must uphold ethical principles, respect intellectual property rights, and transparency when accessing traditional knowledge and resources. Ethnopharmacological research demands the establishment of benefit-sharing agreements that honor the contributions of indigenous communities. Collaborations should be characterized by equitable distribution of benefits, including financial compensation, capacity-building initiatives, and opportunities for community empowerment. These agreements not only respect the intellectual property of traditional knowledge holders but also foster mutual respect, cultural preservation, and socioeconomic development within the originating communities [158].

The journey from traditional knowledge to evidence-based interventions requires harmonizing scientific rigor, ethical principles, and cultural respect. Collaborative efforts between researchers, healthcare practitioners, and indigenous communities must be founded on mutual trust and recognition. Transparency in research processes, adherence to ethical guidelines, and open dialogues surrounding benefit-sharing agreements are essential to ensure that the promise of ethnopharmacologic interventions against PD is realized while avoiding exploitation and misappropriation. While ethnopharmacologic interventions for PD bring forth the potential to enrich therapeutic options, the path is replete with challenges. The balance between preserving cultural integrity and translating traditional wisdom into evidence-based treatments is complex. Standardization, quality control, ethical considerations, and benefit-sharing mechanisms emerge as critical cornerstones in this journey, a journey that, when navigated thoughtfully and ethically, holds the potential to honor indigenous contributions and advance the fight against PD [159].

## 13 PRECLINICAL AND CLINICAL EVIDENCE

The realm of ethnopharmacologic interventions for PD is characterized by a two-fold exploration encompassing preclinical studies conducted *in vitro* and on animal models and clinical trials involving human subjects. This holistic approach delves into the potential therapeutic efficacy of natural compounds and herbal formulations. The convergence of these preclinical and clinical investigations not only elucidates the promise of ethnopharmacologic interventions but also highlights the complexities and challenges in translating findings from bench to bedside. Preclinical studies, often initiated *in vitro*, serve as the initial stepping stones to elucidate ethnopharmacologic interventions' potential mechanisms and effects. These studies frequently employ cellular models to decipher the impact of natural compounds on relevant molecular pathways implicated in PD pathophysiology. For instance, the evaluation of curcumin's anti-inflammatory effects or the impact of resveratrol on oxidative stress pathways may occur in these controlled environments [160, 161].

Building upon these *in vitro* insights, animal models bridge cellular findings and potential clinical applications. Rodent models, such as the 6-hydroxydopamine (6-OHDA) and 1-methyl-4-phenyl-1,2,3,6-tetrahydropyridine (MPTP) models, simulate aspects of PD pathology, enabling the investigation of natural compounds and herbal formulations in a living system. These animal studies offer a comprehensive view of interventions' effects on motor behavior, biochemical markers, histopathological changes, and neuroinflammation. They contribute to a nuanced understanding of potential efficacy, optimal dosing, and safety profiles [160, 162].

Clinical trials constitute a pivotal stage in the journey from experimental evidence to therapeutic validation. These trials rigorously assess the safety and efficacy of ethnopharmacologic interventions in human subjects. Clinical studies encompass various phases, including Phase I (safety), Phase II (efficacy), and Phase III (large-scale efficacy and safety). During these phases, participants are administered the natural compounds or formulations, and outcomes such as motor function improvement, cognitive enhancements, or quality of life assessments are evaluated. Methodologies within clinical trials involve randomized, double-blinded, placebo-controlled designs to minimize bias and ensure robust results. Selecting appropriate outcome measures, such as Unified PD Rating Scale (UPDRS) scores or neuroimaging analyses, enhances the accuracy of assessing intervention effects. Clinical trials also explore potential side effects, drug interactions, and long-term safety profiles [163, 164]. Despite their significance, clinical trials also harbor limitations. Small sample sizes, placebo effects, patient heterogeneity, and the variability in disease progression may influence trial outcomes. Ethnopharmacological interventions can present additional challenges, including variations in traditional preparation methods, regional differences in plant constituents, and complex interactions within multi-ingredient formulations. The confluence of preclinical investigations and clinical trials provides a comprehensive view of ethnopharmacologic interventions' potential in PD management. Successful outcomes in preclinical studies pave the way for human trials, where the transferability of findings from bench to bedside is tested. However, the journey is full of challenges. The intricate web of molecular pathways, individual variability, and the complexities of PD's multifaceted nature contribute to the intricate landscape of preclinical and clinical studies, exploring ethnopharmacologic interventions for PD journeys from early preclinical insights to the rigor of clinical trials. *In vitro* and animal model studies lay the foundation by uncovering potential mechanisms and effects, while clinical trials shed light on safety, efficacy, and challenges in human subjects. Though accompanied by complexities, this holistic approach promises to bridge the gap between traditional wisdom and evidence-based medicine, ultimately contributing to improving PD management strategies [165, 166].

## 14 COMPUTATIONAL METHODS IN NEUROPHARMACOLOGICAL RESEARCH FOR PARKINSON'S DISEASE

Computational methods play a pivotal role in advancing our understanding of Parkinson's disease (PD) and the development of neuropharmacological interventions derived from plant sources. These methods encompass a range of techniques aimed at elucidating the underlying mechanisms of PD pathophysiology, predicting drug-target interactions, and optimizing therapeutic strategies. In the context of geriatric psychiatry, computational approaches offer valuable insights into the complexities of PD progression and its impact on mental health in elderly populations.

### 14.1 Molecular Docking Studies

Molecular docking serves as a cornerstone in neuropharmacological research by predicting the binding interactions between small molecules (*i.e*., plant-derived compounds) and target proteins implicated in PD pathology. Utilizing algorithms such as AutoDock and Vina, researchers can simulate the conformational flexibility of ligands and receptors to identify potential drug candidates with high binding affinity and specificity. In the context of PD, molecular docking studies facilitate the screening of natural compounds for their ability to modulate key molecular targets, including dopamine receptors, monoamine oxidase inhibitors (MAOIs), and α-synuclein aggregates, thereby elucidating their therapeutic potential in ameliorating motor and non-motor symptoms associated with PD.

### 14.2 Molecular Dynamics Simulations

Molecular dynamics (MD) simulations offer a dynamic perspective on ligand-receptor interactions by simulating the trajectories of atoms and molecules over time. By integrating principles of classical mechanics and statistical thermodynamics, MD simulations provide insights into the structural dynamics and stability of protein-ligand complexes, as well as the underlying mechanisms of ligand-induced conformational changes. In the context of PD, MD simulations enable researchers to explore the dynamic behavior of dopaminergic receptors, α-synuclein fibrils, and other molecular targets implicated in disease pathogenesis, thereby informing the rational design of novel neuroprotective agents derived from plant sources.

### 14.3 Pharmacophore Modeling and Virtual Screening

Pharmacophore modeling involves the identification of key molecular features essential for ligand binding and biological activity. By constructing three-dimensional models of pharmacophore hypotheses based on known ligand-receptor interactions, researchers can conduct virtual screening of compound libraries to prioritize lead compounds with optimal pharmacological profiles. In the context of PD, pharmacophore modeling enables the identification of structurally diverse plant-derived compounds that exhibit complementary binding interactions with dopaminergic receptors, antioxidant enzymes, and neuroinflammatory mediators implicated in PD pathogenesis.

### 14.4 Network Pharmacology and Systems Biology

Network pharmacology approaches leverage computational algorithms and network analysis techniques to elucidate the complex interactions between drugs, targets, and biological pathways underlying PD pathophysiology. By integrating data from diverse omics platforms (*e.g*., genomics, proteomics, and metabolomics), network pharmacology enables the construction of disease-specific interaction networks and the identification of novel drug targets and synergistic drug combinations. In the context of geriatric psychiatry, network pharmacology holds promise for unraveling the multifactorial etiology of PD-related neuropsychiatric symptoms and identifying plant-derived interventions with pleiotropic effects on neuronal function and synaptic plasticity.

The computational methods serve as indispensable tools in neuropharmacological research for Parkinson's disease, facilitating the discovery and optimization of plant-derived therapeutics targeting key molecular pathways implicated in disease pathogenesis. By harnessing the power of computational modeling and simulation techniques, researchers can accelerate the translation of preclinical findings into clinically efficacious interventions for geriatric psychiatry.

## 15 FUTURE DIRECTIONS AND IMPLICATIONS

As ethnopharmacological research continues bridging the chasm between traditional wisdom and contemporary scientific inquiry in PD, the landscape unveils promising vistas for future directions and implications. This voyage of discovery underscores the integration of diverse disciplines and envisions the fusion of indigenous knowledge with modern scientific methodologies. The road ahead leads to personalized medicine, biomarker exploration, and the emergence of innovative therapeutics that stand to revolutionize PD management. The future trajectory of ethnopharmacologic research in PD rests upon the shoulders of multidisciplinary collaborations. By uniting traditional knowledge bearers, ethnobotanists, pharmacologists, neuroscientists, geneticists, and clinicians, a holistic perspective emerges that transcends the boundaries of individual disciplines. These collaborations foster cross-fertilization of insights, amplifying the potential for novel discoveries and comprehensive interventions. The merging of indigenous wisdom with modern scientific rigor encapsulates a harmonious symphony that enriches the pursuit of effective PD management. The journey forward hinges upon integrating traditional knowledge with cutting-edge scientific methodologies. Harnessing the insights derived from ethnopharmacologic studies, researchers can design experiments that validate the efficacy of traditional treatments and unravel their underlying mechanisms. Techniques such as metabolomics, transcriptomics, and neuroimaging offer windows into the intricate tapestry of biological responses, complementing the holistic understanding of indigenous practices.

The era of personalized medicine beckons, where ethnopharmacologic interventions can be finely tuned to match each individual's unique genetic makeup, environmental influences, and disease progression. By leveraging genomics, proteomics, and other omics technologies, treatments can be tailored to specific subtypes of PD, optimizing therapeutic outcomes and minimizing side effects. This personalized approach ensures that the interventions resonate harmoniously with the intricate dance of each patient's physiology. Ethnopharmacologic research paves the way for biomarker discovery, unlocking pathways that enable early detection and precise monitoring of PD. Indigenous treatments may be vital to unveiling novel biomarkers that signal disease onset, progression, or response to therapy. Such biomarkers, rooted in traditional practices, can usher in a new era of timely interventions, mitigating the impact of PD before it reaches advanced stages. Ethnopharmacologic research tantalizes with the promise of novel therapeutics that extend beyond conventional approaches. Integrating traditional plant-based remedies with modern drug discovery methodologies offers a treasure trove of natural compounds waiting to be harnessed. As observed in indigenous formulations, the synergy between compounds presents opportunities to develop innovative therapies that target multiple facets of PD's complex pathophysiology. The future directions of ethnopharmacologic research in PD epitomize the synergy between tradition and innovation. As diverse disciplines converge, integrating traditional wisdom with modern science unfurls a tapestry of possibilities. Collaborations echo the harmonious melody of multifaceted expertise while personalized medicine, biomarker discovery, and novel therapeutics are progress's guiding stars. The horizon beckons, a realm where the rich legacies of ancestral knowledge intertwine with the frontiers of contemporary discovery, nurturing a transformational journey toward enhanced PD management.

## 16 CHALLENGES AND CONSIDERATIONS

The journey of developing novel treatments for PD is riddled with challenges that demand meticulous attention, from the design of clinical trials to regulatory hurdles and vigilance against potential adverse effects. These complexities underscore the necessity of a balanced risk-benefit assessment to ensure the safety and efficacy of emerging interventions, harmonizing the pursuit of innovative solutions with the safeguarding of patient welfare.

### 16.1 Clinical Trial Design: A Delicate Balancing Act

Designing clinical trials for PD interventions requires a delicate balance between scientific rigor and patient well-being. Selecting appropriate endpoints, such as motor function improvement, quality of life enhancement, or biomarker modulation, is pivotal to assessing intervention outcomes accurately. The challenges lie in the variability of PD's clinical presentation, the fluctuating nature of symptoms, and the potential placebo effects. Researchers must deploy sophisticated statistical methodologies to discern actual treatment effects from background noise and fluctuations, ensuring that trial results accurately reflect the interventions' efficacy.

### 16.2 Regulatory Hurdles: Navigating the Path to Approval

The landscape of regulatory approvals for PD therapeutics is fraught with rigorous standards that ensure patient safety and treatment efficacy. New interventions, whether derived from traditional knowledge or modern drug discovery, must adhere to stringent guidelines set forth by regulatory bodies like the Food and Drug Administration (FDA) and the European Medicines Agency (EMA). These guidelines encompass preclinical data, clinical trial protocols, safety profiles, and manufacturing processes, necessitating comprehensive documentation and meticulous adherence to ethical and scientific principles.

### 16.3 Potential Adverse Effects: The Challenge of Unforeseen Consequences

Introducing new treatments, regardless of their origins, carries the inherent risk of potential adverse effects. The intricacies of PD's pathophysiology and the complex interactions of interventions within the human body may lead to unforeseen consequences. These adverse effects could manifest as drug interactions, exacerbation of pre-existing conditions, or entirely new physiological responses. Researchers must remain vigilant, closely monitoring trial participants for any untoward effects and being prepared to address them while swiftly maintaining ethical obligations to participant well-being.

### 16.4 Balanced Risk-Benefit Assessment: The Linchpin of Ethical Progress

The cornerstone of ethical drug development lies in the delicate equilibrium of risk and benefit assessment. Researchers must meticulously weigh the potential benefits of novel interventions against their associated risks, both known and unknown. The informed consent process becomes paramount, as participants must be comprehensively educated about potential adverse effects, ensuring their autonomy and informed decision-making. A balanced risk-benefit assessment safeguards patients from undue harm while fostering an environment conducive to innovative therapeutic advancements.

### 16.5 Ethical Dimensions and Patient Welfare

In essence, the pursuit of new treatments for PD ushers forth a landscape of multifaceted challenges. The intricacies of clinical trial design, regulatory navigation, and the anticipation of potential adverse effects are critical considerations in this journey. The ethical dimensions of patient welfare underscore the need for a balanced risk-benefit assessment that honors scientific innovation while placing patient safety at the forefront. Harmonizing these facets encapsulates a responsible and conscientious approach that seeks to elevate PD management while upholding the highest standards of ethical conduct.

## CONCLUSION

Parkinson’s disease (PD) poses a significant clinical and scientific challenge due to its multifactorial etiology, progressive nature, and the coexistence of both motor and non-motor symptoms. While conventional pharmacotherapy, including levodopa and dopamine agonists, remains the cornerstone of symptom management, these approaches are often limited by long-term complications and insufficient efficacy against non-motor manifestations. In light of these limitations, there is a growing interest in the integration of ethnopharmacological knowledge and modern scientific methodologies as a complementary approach to PD management.

This review highlights the therapeutic potential of traditional herbal formulations, notably Mucuna pruriens, Bacopa monnieri, curcumin, resveratrol, and various flavonoids, which exhibit diverse neuroprotective effects mediated through antioxidative, anti-inflammatory, and anti-apoptotic mechanisms. Moreover, incorporating computational tools, such as molecular docking, pharmacophore modeling, and network pharmacology, has enabled a deeper understanding of their mechanisms of action, target interactions, and synergistic effects. These technologies enhance the predictive accuracy of drug efficacy and contribute to the rational design of multi-target therapeutic strategies.

Furthermore, the concept of synergism in multi-herbal formulations resonates with the pathophysiological complexity of PD, addressing multiple disease pathways simultaneously. However, despite promising preclinical and preliminary clinical evidence, considerable challenges remain, including standardization, quality control, regulatory validation, and the ethical inclusion of indigenous knowledge systems.

In conclusion, the integration of traditional medicine with computational pharmacology presents a novel, holistic, and potentially transformative framework for the development of multi-target therapeutics in PD. Future research must emphasize rigorous clinical validation, cross-disciplinary collaboration, and ethical stewardship to translate these insights into evidence-based, patient-centered interventions.

## Figures and Tables

**Table 1:** Ethnopharmacologic approaches for Parkinson's disease related symptoms.

**Scientific Name (Family)**	**Local Name**	**Medicinal Use**	**Part(s) Used**
*Ananas comosus* (L.) Merr. (Bromeliaceae)	Pineapple	Anti-inflammatory	Fruits
Arnica species (Asteraceae)	Arnica	Analgesic	Roots
*Brassica oleracea* L. (Brassicaceae),	Cabbage	Anti-inflammatory	Leaves
*Eupatorium perfoliatum* L. (Asteraceae)	Boneset	Analgesic	Bark
*Hypericum perforatum* L. (Hypericaceae)	St John's Wort	Antidepressant	Bark
Lavandula species (Lamiaceae)	Lavender	Soporific	Flowers
*Melissa officinalis* L. (Lamiaceae),	Lemon balm	Sedative	Leaves
Mentha species (Lamiaceae)	Mint	Sedative	Leaves
*Rosmarinus officinialis* L. (Lamiaceae),	Rosemary	Good for nerves, stimulant	Leaves
*Sambucus nigra* L. (Adoxaceae)	Elderberry	Analgesic, sedative	Flowers and berries
*Sassafras albidum* (Nutt.) (Lauraceae)	Sassafras	Anxiety treatment	Roots
*Taraxacum officinale* (L.) (Asteraceae)	Dandelion	Digestive disorders	Roots and Leaves
*Valeriana officinalis* L. (Caprifoliaceae),	Valerian	Soporific	Roots
*Verbascum thapsus* L. (Scrophulariaceae)	Mullein	Analgesic	Leaves and Roots

## LIST OF ABBREVIATIONS

AADC: Aromatic L-Amino Acid Decarboxylase
AChEIs: Acetylcholinesterase Inhibitors
COX-2: Cyclooxygenase-2
DJ-1: Protein Deglycase DJ:1 Gene
EGCG: Epigallocatechin Gallate
ER: Extended-Release
IL-1β: Interleukin-1beta
iNOS: Inducible Nitric Oxide Synthase
L-DOPA: Levodopa
LRRK2: Leucine-rich Repeat Kinase 2 Gene
MAO-B Inhibitors: Monoamine Oxidase Type B Inhibitors
MAO-B: Monoamine Oxidase-B
MCI: Mild Cognitive Impairment
NF-κB: Nuclear Factor-kappa B
NMDA: N-Methyl-D-Aspartate
NMS: Non-motor Symptoms
Nrf2: Nuclear Factor Erythroid 2-related Factor 2
PARKIN: Parkin RBR E3 Ubiquitin-protein Ligase Gene
PD: Parkinson's Disease
PGC-1α: Peroxisome Proliferator-activated Receptor-gamma Coactivator-1 Alpha
PINK1: PTEN-induced Kinase 1 Gene
RNS: Reactive Nitrogen Species
ROS: Reactive Oxygen Species
SNCA: Alpha-synuclein Gene
SNRIs: Serotonin-norepinephrine Reuptake Inhibitors
SOD: Superoxide Dismutase
SSRIs: Selective Serotonin Reuptake Inhibitors
TCM: Traditional Chinese Medicine
TNF-α: Tumor Necrosis Factor-alpha
